# Annotations

**Published:** 1898-04-02

**Authors:** 


### Annotations.

### Deaths from Vaccination.

The result of an inquest held last week at Battersea on three children who had died from erysipelas following vaccination was the passing of a verdict which, while not affecting the general question of vaccination, condemned very strongly the manner in which it was carried out in these particular cases. According to the summing-up of the coroner (Mr. Braxton Hicks) the deceased, together with a fourth child named Woods, developed symptoms of erysipelas within 24 hours of being vaccinated with lymph taken from the arm of a child named Atkinson, who already had a skin affection, and he added that the system admittedly adopted by the public vaccinator of blowing lymph from tubes on to his thumb nail was indefensible. The finding of the jury showed that the vaccination was not carried out according to the instructions of the Local Government Board, that the vaccinifer and the vaccinees were not fully examined, and that the lancet was not properly cleansed before each operation. This is a matter of great public importance. It would, indeed, have been a matter of very serious gravity if such an outbreak of erysipelas had been shown to have occurred among cases which had been properly vaccinated with lymph collected in accordance with the instructions under which the general store of lymph is got together, and used in accordance with the directions in accordance with which the compulsory vaccination of the population has to be performed. We must, however decline to recognise as vaccination an operation done with lymph derived from a diseased child and used in a manner the reverse of cleanly. Those who have watched the enormous strides made in surgery during recent years, and have noted how on every hand such improvements have gone hand in hand with the exercise of more and more care in the avoidance of dirt, cannot fail to see that in the hands of many operators vaccination has sadly lagged behind other surgical procedures. In pathological laboratories the art of vaccination has been perfected to such a degree that its results can be depended on with almost scientific precision. This, however, is merely because vaccination for scientific objects is performed with the antiseptic precautions necessary to ensure proper and uncomplicated results. It ought to be possible to do the same with children.

### Feeble-minded Children.

The committee which has been sitting since December, 1896, for the purpose of inquiring into the existing systems of education of feeble-minded children, has finished its work and sent out its report. The recommendations of the committee show that they fully appreciate the difficulty of differentiating feebleness of mind from mere backwardness of development on the one hand and imbecility on the other. For this reason they recommend that no child should be placed in a special class for weak-minded children before the age of seven years. Up to that age the feeble mind can hardly be diagnosed with assurance, for many quite normal children are backward in their earlier years; and, moreover, the ordinary infant school, with its large amount of kindergarten exercises, is as good training for a feeble-minded child of that age as can be. After that age admission into a special school may be asked on behalf of the child by the teacher of the ordinary school, where so far he has been educated. For the work of teaching in the special schools for the feeble-minded, the committee insist on the need for both training in and love for the work. No school intended for these special cases should contain more than 120 children, and no class more than 20. The lessons should be short, and great latitude should be allowed in following the time-table, as the children will require to be individually dealt with, and there will be greater variations of temper and capacity than are to be looked for among normal children. The committee also dwell on the value of manual training in the curriculum of the special schools, as the facts that they have gathered seem to indicate that hand-work helps brain development more than head-work does in the case of these children. The number of feeble-minded children is comparatively small, only 1 per cent. of the whole school-going population, and this proportion seems to be practically invariable, for the committee found it to be the same not only throughout Britain, but in the other countries from which they drew information. Therefore, except in the large towns, schools would be few, and scattered over large areas. This makes it desirable that school authorities should, with the consent of the parents, have the power to board out children in the neighbourhood of special schools, and to send children to certified homes in connection with them, or to provide guides and conveyances to the school, as the nature of the case may render advisable.

### The Population Question.

The *Humanitarian* for April has another of those curious articles which have been so common of late—articles railing at modern marriages and modern methods of giving in marriage, and suggesting all sorts of dreadful fates for those who are not considered worthy to be fathers of the race. We cannot but feel, however, that although it may be very comforting and may sound very scientific to anticipate a time "when the population of the earth shall be kept as strictly within the bounds of number and suitability of race as the sheep on a well-ordered moor," the authors of these ingenious suggestions ought to make some specific proposal, instead of airing their more or less pious opinions. How is marriage to be limited without immorality being extended; how are the criminals, tramps, paupers, and vagrants, and the children and grandchildren of parents possessing taints of insanity, idiocy, intemperance, cancer, tuberculosis, deformity, and suicide, to be kept from propagating their kind? It is said "could the fear of being made sexless be held over the claimants for the charity of a Salvation Army shelter, a 'Barnardo Home,' or a reformatory, what an effect it would have." This is all very fine, but is it not strongly tainted with a savour of nonsense? If we find it so difficult now to decide who is a fit object of charity, how are we to decide what amount of criminality or vagrancy shall condemn a man to the knife, or what degree of defect in a girl's grandmother shall subject her to oophorectomy? Let us have a scheme, and then we will discuss it.

